# Modelling and Analysis of Characteristics of a Piezoelectric-Actuated Micro-/Nano Compliant Platform Using Bond Graph Approach

**DOI:** 10.3390/mi9100498

**Published:** 2018-09-29

**Authors:** Chao Lin, Zhonglei Shen, Jiang Yu, Pingyang Li, Dehong Huo

**Affiliations:** 1 State Key Laboratory of Mechanical Transmission, Chongqing University, Chongqing 400044, China; szlemail@163.com (Z.S.); 20160702068t@cqu.edu.cn (J.Y.); 595192166lpy@gmail.com (P.L.); 2 School of Mechanical and Systems Engineering, Newcastle University, Newcastle upon Tyne NE1 7RU, UK; dehong.huo@newcastle.ac.uk

**Keywords:** micro-/nano compliant platform, piezoelectric actuator, bridge-type displacement amplification mechanism, bond graph

## Abstract

The piezoelectric-actuated flexure-based compliant platform is commonly adopted in many fields of micro and nanotechnology. In this paper, bond graph modeling, and kinematic and dynamic characteristics of a piezoelectric-actuated micro-/nano compliant platform system are investigated. During modeling, the bond graph model of the piezoelectric actuator (PZT) is derived by considering both the electrical domain and the mechanical domain. Considering the compliances of flexure hinges and elastic linkages, as well as the input ends, the bond graph model for the bridge-type displacement amplification mechanism in the compliant platform is established by combining pseudo-rigid-body (PRB) model theory and elastic beam theory. Based on the interactions between the PZT subsystem and compliant platform subsystem, the kinematic performance of the proposed compliant platform system is evaluated through both computer simulations and experimental tests. Furthermore, the frequency responses, dynamic responses and load capacity of the compliant platform system are studied. This paper explores a new modeling method for a piezoelectric-actuated compliant platform system, which can provide an effective solution when analyzing the micro-/nano system.

## 1. Introduction

With the rapid development of micro-/nanotechnology, piezoelectric-actuated, flexure hinge-based compliant platforms are now used across a wide spectrum of fields, including nanopositioning systems [1,2], ultra-precision manufacturing [3,4], scanning probe microscopes [5], and biomedical cell micro-manipulation [6,7]. The reasons for the promise of these applications are mainly attributed to the combination of the piezoelectric actuator (PZT) and flexure hinge-based compliant mechanisms. The PZT with its characteristics of high resolution, large output force, high stiffness and fast dynamic response is also applied in many other technological fields such as fluid jetting dispensers [8,9] and active shape control for aircraft [10,11]. However, its drawback is that the stroke of the PZT is inherently small. Thus, flexure hinge-based displacement amplification mechanisms [12,13,14] with the merits of being without friction wear and backlash, are frequently designed to magnify the output displacement of the PZT. In particular, the bridge-type mechanism [15,16] has been widely used as a basic element to construct a more complex compliant platform with multi-degrees of freedom [17,18,19], due to its compact structure and large displacement amplification ratio.

To obtain a piezoelectric-actuated compliant platform system with better performance and design a controller, it is necessary to predict the kinematic and dynamic characteristics by considering both the PZT and the compliant platform. Previous research has derived a variety of mathematic models for describing the characteristics of the PZT. Goldfarb et al. [20] proposed a model in which a lumped-parameter energy-based representation was introduced to describe the static and dynamic behaviors of the PZT, which has been widely used for modeling the piezoelectric-actuated positioning system [21,22]. Rodriguez-Fortun et al. [23] presented a mathematic model for describing both hysteresis and rate dependence of the PZT, which took into account three coupled physical domains, the electric domain, the material domain and the mechanical domain. In addition, the well-known pseudo-rigid-body (PRB) model was proposed by Howell et al. [24] to address static, kinematic and dynamic analysis of the compliant mechanisms for a well-designed mechanism, which approximates a compliant mechanism as an equivalent rigid-body mechanism, and then the rigid-link mechanism theory can be applied to analyze the compliant mechanism. Based on PRB model theory, many analytical modeling methods have been developed over the past decade for static and kinematic analysis of compliant mechanisms by combining the matrix method [2,18,19], elastic beam theory [14,25] and Castigliano’s second theorem [1]. Moreover, the dynamic modeling approaches of compliant mechanisms were also studied by combining PRB model theory and Lagrange’s equation [26,27]. She et al. [28] investigated the dynamics of compliant mechanisms by introducing a set of non-dimensional mass property parameters to the PRB model. Li et al. [29] studied the dynamics of the PRB model considering the mass parameters. In addition, the finite element method (FEM) is also an approach commonly used to analyze compliant mechanisms [30,31], although the FEM requires a large computational load to obtain acceptable accuracy. Furthermore, some scholars have begun to study a generalized modeling method using interactions between PZTs and compliant mechanisms. Ryu et al. [32] proposed a kinematic and dynamic modeling method by assuming both the PZT and the compliant mechanism to be a spring-mass system. Tian et al. [12] investigated the dynamic performance through interactions between the PZT and the compliant mechanism. Gu et al. [21] presented a general model to represent the dynamic behaviors of both the PZT and the compliant mechanism. As described above, the piezoelectric-actuated compliant platform is a type of multi-energy domain system involving mechanical and electrical energy domains. However, previous research about the interaction between the PZT and the compliant mechanism has always focused on one side (either the electrical part or the mechanical part) and simplified the other too much. In addition, a co-simulation approach is commonly used for obtaining the simulated results; such as a mechanical model in finite element analysis and an electrical model in Matlab/Simulink [33]. However, the main issue, in the co-simulation, is the trade-off between calculation efficiency and accuracy. Thus, a major challenge in the study of a piezoelectric-actuated compliant system is to generate a unified model that contains a multi-energy domain subsystem and can deduce uniform algebraic relations among state variables.

Bond graph theory is a general modeling method proposed by Henry Paynter from MIT. The bond graph model represents all types of physical systems by considering the power exchange between its unified basic elements [31]. Thus, the bond graph approach is quite suitable for modeling the interaction between the different multi-energy domain subsystems [31,34]. As described above, the proposed piezoelectric-actuated compliant platform system is a typical multi-energy domain system involving electronic, piezoelectric and mechanical energy domains. Some research institutes have begun to build bond graph models for piezoelectric actuators [23,35] and compliant mechanisms [36,37] Nevertheless, there are few studies which have focused on bond graph modeling of integral piezoelectric-actuated compliant mechanism systems [8,38], which remains to be further developed.

In this study, the mechatronics model of a piezoelectric-actuated micro-/nano compliant platform system is established based on the bond graph approach. By simulating the bond graph model, the kinematic and dynamic performances of the interactions between the PZT and the compliant platform are investigated. In addition, the performance and effectiveness of the established bond graph model is verified by experimental tests. The remainder of this paper is organized as follows: The structure description and working principles of the compliant platform are introduced in Section 2; Section 3 establishes the bond graph model of the compliant platform system through interactions between the mechanical subsystem and the electrical subsystem; In Section 4, the kinematic and dynamic performances of the compliant platform are obtained by simulations and experiments; and finally, conclusions are reached in Section 5.

## 2. Structure Description

**Figure 1:**
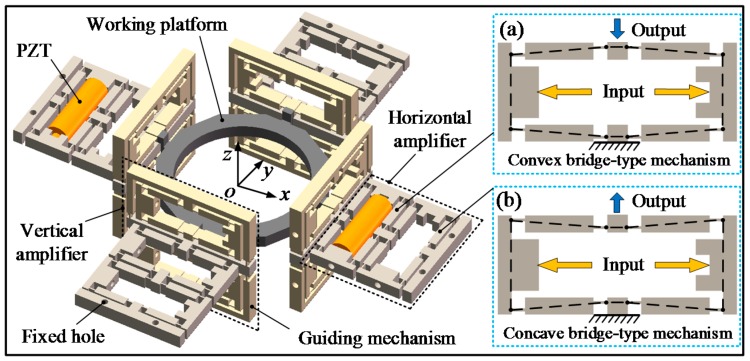
Schematic diagram of the piezoelectric-actuated compliant platform.

Figure 1 shows the schematic representation of the piezoelectric-actuated micro-/nano compliant platform system. As shown in Figure 1, the proposed compliant platform system is constructed with the PZTs for actuation as well as the compliant platform for motion transmission. To avoid undesirable cross-axis coupling motions, the structure of the compliant platform was designed symmetrically, and its length, width and height are 266 mm × 266 mm × 82 mm. Due to the small working range of the PZT, the bridge-type displacement amplification mechanism is employed to magnify the out displacement of the PZT; its total dimensions are 70 mm × 29 mm × 8 mm and the amplified displacement can be obtained through the working platform. The compliant platform consists of four horizontal amplifiers to realize movement along the *X*/*Y* direction, and four vertical amplifiers for moving in the *Z* direction and rotating around the *X*/*Y* direction. Both the horizontal amplifier and vertical amplifier constitute two bridge-type displacement amplification mechanisms with the same geometric parameters. The right-angle flexure hinges are adopted owing to the large compliance in the rotational direction and large stiffness in the cross-axis coupling direction. The PZT is inserted inside the bridge-type mechanism to generate an input displacement. In order to obtain a larger motion stroke in the *X* and *Y* directions, each of the horizontal amplifiers include a convex bridge-type mechanism (Figure 1a) and a concave bridge-type mechanism (Figure 1b). Both of them have the same working principle, except that the motion directions of the output end are different, as is commonly used in the flexure-based compliant platform [17]. Similarly, each of the vertical amplifiers consists of two of the convex bridge-type mechanisms to obtain a larger travel range. Due to the symmetric structure of the compliant platform in the horizontal direction, the same performances are obtained in the *X* and *Y* directions. To avoid undesirable parasitic motions, the parallelogram guiding mechanisms are employed to guide the motion of the working platform in the *Z* direction and to provide fixed constraints for the bridge-type mechanism as shown in Figure 1.

During operation, when a convex bridge-type mechanism and the concave bridge-type mechanism on the opposite side in the horizontal amplifier are working simultaneously, the working platform achieves movement along the *X*/*Y* direction. When the four bridge-type mechanisms of the bottom vertical amplifier are working simultaneously, movement along the forward direction of the *Z* direction is obtained, while the upper four bridge-type mechanisms of the vertical amplifier work together to obtain motion in the opposite direction. When only two of bridge-type mechanisms in the diagonal direction of the vertical amplifier are working together, the rotation motion around the *X*/*Y* direction can be obtained.

## 3. Bond Graph Model of the Compliant Platform System

As depicted in Figure 1, the compliant platform system consists of the piezoelectric part and the mechanical part. When a driving voltage is applied to the PZT, it can generate an output displacement and output force and act as the input of the mechanical part. The output displacement of the PZT is magnified through the bridge-type displacement amplification mechanism, and the amplified displacement is outputted through the working platform. In this study, the modular and assembled modeling strategy is adopted to build the bond graph model of the compliant platform system. In Section 3.1 and Section 3.2, the PZT and bridge-type displacement amplification mechanism are considered as basic units of the compliant platform and separately modeled. They are then coupled together in series or parallel by adding some signal flows and power bonds to obtain the bond graph model of the whole compliant platform system. 

### 3.1. Bond Graph Model of the Piezoelectric Actuator

**Figure 2:**
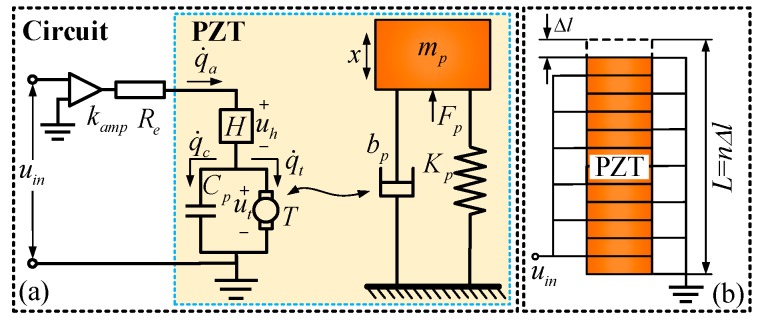
Schematic diagram of the piezoelectric actuator: (**a**) Electromechanical model of the piezoelectric actuator; (**b**) connection of the layers comprising the piezoelectric actuator.

Following previous research results that modeled the PZT [20], the multi-domain model for representing a PZT is illustrated by Figure 2a, and both the electrical and the mechanical domains are described. 

In the electric domain, the inverse piezoelectric effect of the piezoelectric material is considered in modeling. The driving circuit of the PZT can be simplified as a voltage amplifier with an amplification ratio of $k_{amp}$ and an equivalent resistance of $R_{e}$, and $u_{in}$ is the input voltage for the PZT. Moreover, $u_{h}$ is the voltage result from the hysteresis effect, and $u_{t}$ is the transduced voltage. *T* is the electromechanical transformation factor, and $C_{p}$ is the capacitance of the whole PZT; $q_{i}\left(i=a,c,t\right)$ are the total input charge, the stored charge of capacitance $C_{p}$ and the transduced charge, respectively. The complete electrical equations can be expressed as follows [21]:

(1) $$R_{e}{\dot{q}}_{a}+u_{h}+u_{t}=k_{amp}u_{in}\;$$

(2) $$q_{a}=q_{c}+q_{t}\;$$

(3) $$q_{c}=u_{t}C_{p}\;$$

(4) $$q_{t}=Tx\;$$

(5) $$u_{t}=q_{c}/C_{p}\;$$

As hysteresis and nonlinearity effect are not the object of this study, only a linear case needs to be considered in dynamic modeling; that is voltage $u_{h}=0$. The capacitance of $C_{p}$ can be treated a linear capacitance on account of ignoring the hysteresis and nonlinearity effect, which can be computed through a linear equation as follows [23]:

(6) $$C_{p}=nc\Delta l^{2}\;$$

where n is the number of piezoelectric layers of the PZT, c is the capacitor value of a single piezoelectric layer, and Δl is the thickness of the single piezoelectric layer as shown in Figure 2b, which are provided by the supplier. As Figure 2b shows, the piezoelectric layers are bonded in series mechanically with each other and their electrodes are connected in such a way that the layers are in parallel electrically.

In the mechanical part, the PZT can be simplified as a mass-spring-damper model with an equivalent mass of $m_{p}$, axis stiffness of $K_{p}$ and an equivalent damping coefficient of $b_{p}$. $F_{p}$ is the transduced force from the electrical side, and x is the output displacement of the PZT. According to Newton’s laws of motion, $F_{p}$ can be derived as:

(7) $$F_{p}=Tu_{t}.$$

**Figure 3:**
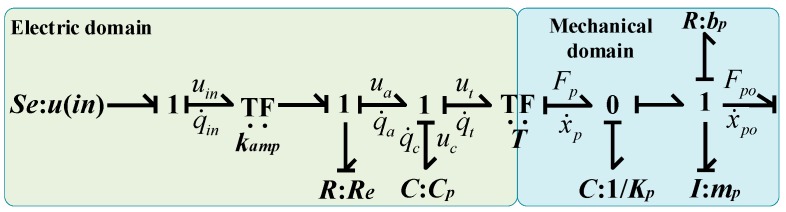
Bond graph model of the piezoelectric actuator.

Based on the analysis above, the bond graph model of the PZT can be modeled as illustrated in Figure 3. The parameters, $F_{po}$ and ${\dot{x}}_{po}$ represent the output force and velocity of the end effector of the PZT. The transformer *TF*, whose value is equal to *T*, represents the model of a power transformation between the electric and the mechanical domain. 

### 3.2. Bond Graph Model of the Bridge-Type Displacement Amplification Mechanism

**Figure 4:**
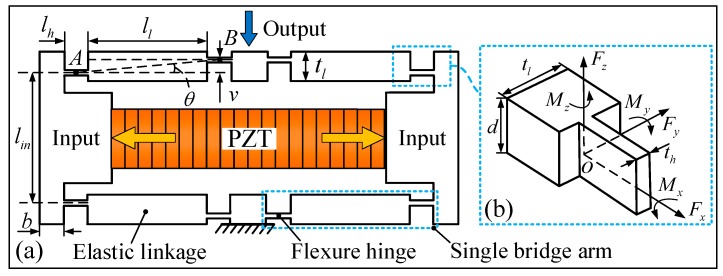
(**a**) Schematic diagram of the bridge-type mechanism, (**b**) coordinate system of right-angle flexure hinge.

**Figure 5:**
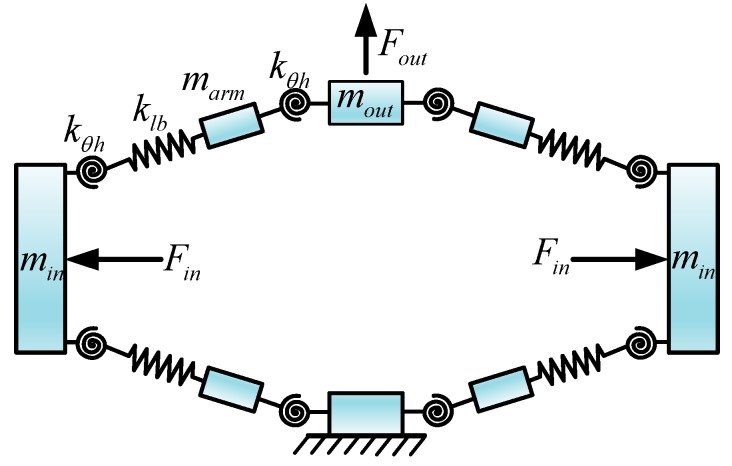
Simplified model of the bridge-type mechanism.

To obtain a desired travel range of the working platform, the bridge-type displacement amplification mechanism is used to magnify the output displacement of the PZT. Figure 4a shows the schematic diagram of a bridge-type displacement amplification mechanism. Once driven by an input displacement/force from the elongation of the PZT, the end effector of the bridge-type mechanism will produce an amplified out displacement. The bridge-type mechanism can have a significant effect on the kinematic and dynamic performance of the compliant platform. To obtain an accurate model, the elastic deformation of the bridge-type mechanism is taken into account in modeling. Because of the double symmetrical structure, only a single bridge arm of the mechanism needs to be analyzed. As depicted in Figure 4a, the single bridge arm is composed of two flexure hinges and an elastic linkage. During the working process of the bridge-type mechanism, the flexible hinge undergoes main bending deformation, so the rotational stiffness of elastic linkage is neglected reasonably, and only the rotational stiffness of flexible hinge is considered. Based on the PRB model, the simplified mass-spring model of the bridge-type mechanism is illustrated in Figure 5, where $F_{in}$ is the input force from the PZT, and $F_{out}$ is the external load applied to the bridge-type mechanism; $k_{\theta h}$ and $k_{lh}$ are the rotational and tensile stiffness of the flexible hinge respectively, and $k_{lb}$ is defined as the tensile stiffness of single bridge arm. 

**Figure 6:**
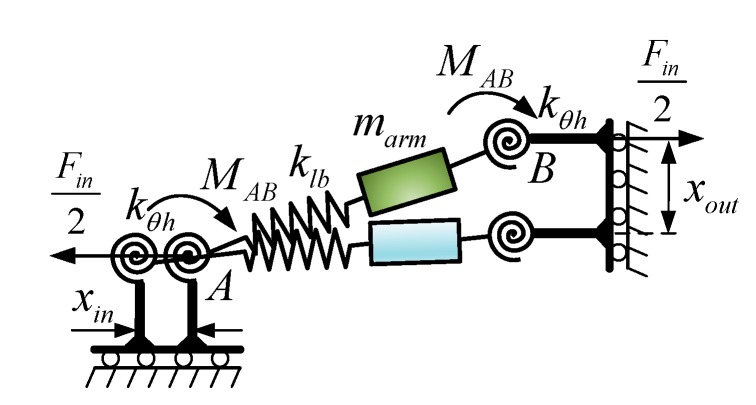
Static model of the single bridge arm.

The static model of the single bridge arm is shown in Figure 6. For single bridge-type mechanisms, it is not necessary to take the external load $F_{out}$ into consideration.

According to the static model as shown in Figure 6, by considering force equilibrium and torque equilibrium, the following relations can be obtained by:

(8) $$\left\{ \begin{array}{l} F_{in}/2=F_{po}/4=F\\ 2M_{AB}=F\left(l_{h}+l_{l}\right)\tan\theta \end{array}\right.\;$$

where $F_{po}$ is the out force of the PZT, and $M_{AB}$ is the torque of flexure hinges; $l_{h}$, $l_{l}$ and θ are structure parameters of the bridge-type mechanism as shown in Figure 4.

When the *x* axis that is aligned along the connecting line of the midpoints *A* and *B* of the adjacent two flexible hinges is assigned, the axis force $F_{AB}\left(x\right)$ and axis torque $M_{AB}\left(x\right)$ of the single bridge arm can be deduced by:

(9) $$\left\{ \begin{array}{l} F_{AB}\left(x\right)=F\cos\theta \\ M_{AB}\left(x\right)=F\times v \end{array}\right.\;$$

where v is the interval between the adjacent two flexible hinges as shown in Figure 4a, which can be calculated as $v=(l_{h}+l_{l})\tan\theta$.

**Figure 7:**
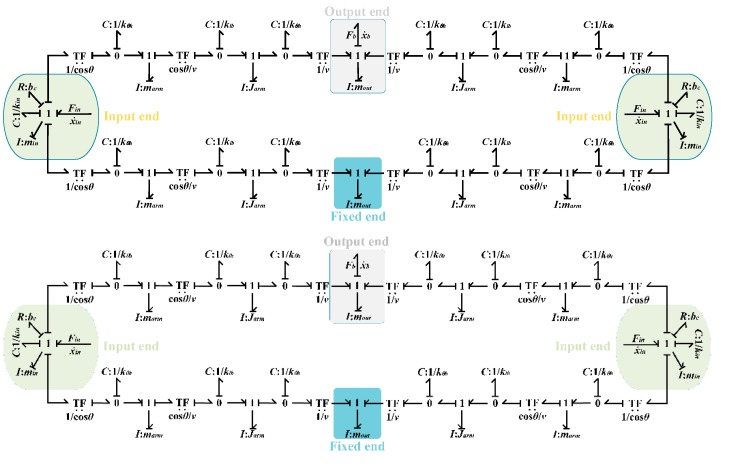
Bond graph model of the bridge-type mechanism.

According to the principle of conservation of energy, the input force from the PZT will be transformed into four parts: the bending deformation energy of flexure hinges and input ends; the tensile deformation energy of the bridge-type arm; the kinetic energy of the bridge-type mechanism; and, the energy consumed by the damping of the mechanical structure. Based on the analysis above and the bond graph technique, the bond graph model of the bridge-type displacement amplification mechanism is derived and depicted in Figure 7.

It can be observed in Figure 7 that the bridge-type mechanism is divided into an input end, output end and fixed end. The driving forces from the PZT are applied to the input ends, and the amplified displacement is obtained by the output end, while the velocity of the fixed end is set to zero. The parameters $F_{in}$ and ${\dot{x}}_{in}$ are the outputs of the PZT model. The parameter $b_{c}$ is the damping parameter of the compliant mechanism. However, the value is hard to obtain before it is identified from experimental results. Hence the damping coefficient will be identified through the experimental data. The transformer *TF*, whose value is equal to 1/cosθ, converts the input force $F_{in}$ of input end into the axis torque $M_{AB}\left(x\right)$ of the flexure hinge. The transformer *TF*, whose value is equal to cosθ/v, converts the axis torque $M_{AB}\left(x\right)$ of the flexure hinge into the axis force $F_{AB}\left(x\right)$ of the single bridge arm. Similarly, the transformer *TF*, whose value is equal to 1/v, converts the axis force $F_{AB}\left(x\right)$ of the single bridge arm into the output force $F_{out}$ of the output end. The parameters $F_{b}$ and ${\dot{x}}_{b}$ represent the output force and output velocity of the bridge-type mechanism. The parameters $m_{in}$, $m_{arm}$ and $m_{out}$ are the mass of the input end, single bridge arm and output end, respectively. The parameter $J_{arm}$ is the rotational inertia of the single bridge arm.

The equivalent tensile stiffness, $k_{lb}$, of the single bridge arm consists of the flexure hinges and elastic linkage. According to the series connection, the equivalent tensile stiffness $k_{lb}$ of the single bridge arm can be derived using:

(10) $$k_{lb}=\frac{2}{k_{lh}}+\frac{1}{k_{ll}}\;$$

where $k_{lh}$ and $k_{ll}$ are the tensile stiffness of flexure hinge and elastic linkage, respectively.

The compliance equation of the flexure hinge is derived from beam theory as follows:

(11) X = CF 

where $X=[\Delta x\;\Delta y\;\Delta z\;\Delta \alpha \;\Delta \beta \;\Delta \gamma {]}^{T},\;F=[F_{x}\;F_{y}\;F_{z}\;M_{x}\;M_{y}\;M_{z}{]}^{T}$.

The compliance matrix of the right-angle flexure hinge can be expressed as Equation (12) [39], which is also suitable for the elastic linkage:

(12) $$C_{i}=[\begin{array}{cccccc} \frac{l_{i}}{Edt_{i}} & 0 & 0 & 0 & 0 & 0\\ 0 & \frac{4l_{i}^{3}}{3Edt_{i}^{3}}+\frac{l}{Gdt_{i}} & 0 & 0 & 0 & \frac{6l_{i}^{2}}{Edt_{i}^{3}}\\ 0 & 0 & \frac{4l_{i}^{3}}{3Ed^{3}t_{i}}+\frac{l}{Gdt_{i}} & 0 & -\frac{6l_{i}^{2}}{Ed^{3}t_{i}} & 0\\ 0 & 0 & 0 & \frac{l}{Gk_{2}dt_{i}{}^{3}} & 0 & 0\\ 0 & 0 & -\frac{6l_{i}^{2}}{Ed^{3}t_{i}} & 0 & \frac{12l_{i}}{Ed^{3}t_{i}} & 0\\ 0 & \frac{6l_{i}^{2}}{Edt_{i}^{3}} & 0 & 0 & 0 & \frac{12l_{i}}{Edt_{i}^{3}} \end{array}]$$

where E and G are the elasticity modulus and shear modulus of material respectively, $k_{2}$ is the geometrical constant determined by $d/t_{i}$, $l_{i}$ and $t_{i}$ are the length and thickness of corresponding structure respectively, and the subscript i=h,l represents the flexure hinge and the elastic linkage, respectively. The coordinate system of the flexure hinge and elastic linkage is shown in Figure 4b. 

The stiffness matrix of the hinge is obtained by the inverse of the compliance matrix as $K_{i}={\left(C_{i}\right)}^{-1}$. Thus, the tensile stiffness $k_{lh}$ and $k_{ll}$, and the rotational stiffness $k_{\theta h}$, can be deduced from Equation (12) as follows:

(13) $$\left\{ \begin{array}{l} k_{lh}=\frac{Edt_{h}}{l_{h}}\\ k_{ll}=\frac{Edt_{l}}{l_{l}}\\ k_{\theta h}=\frac{Edt_{h}{}^{3}}{12l_{h}} \end{array}\right.\;$$

where d, $t_{h}$ and $t_{l}$ are the geometric parameters of the bridge-type mechanism, as shown in Figure 4b.

The input end of the bridge-type mechanism can be simplified as a simply-supported beam. According to Euler-Bernoulli beam theory, the equivalent stiffness, $k_{in}$, of the input end of the bridge-type mechanism can be calculated as the following equation [25]:

(14) $$k_{in}=\frac{48EI}{l_{in}^{3}}\;$$

where *I* is the moment of inertia of the beam, which can be computed as: $I=bd^{3}/12$.

### 3.3. Bond Graph Model of the Compliant Platform System

**Figure 8:**
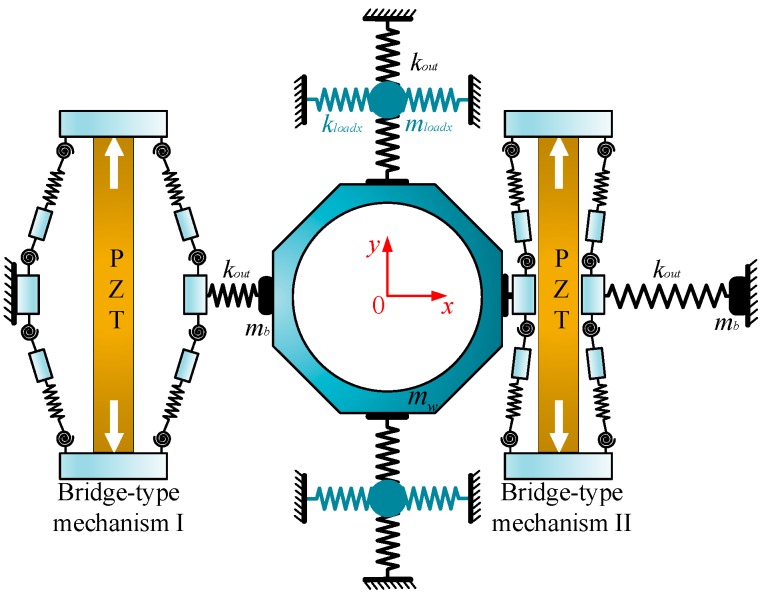
Mechanics model of the compliant platform in the X direction.

The advantage of bond graph modeling is that it can be directly derived according to the physical system, avoiding the derivation of complex mathematical models. Next, a unified bond graph model is constructed for describing all motion of the compliant platform. During operation, the different degrees of freedom have different contributing structures and load stiffness. Therefore, before building the bond graph model of the compliant platform system, the mechanics model in the *X* direction is established as in Figure 8. As depicted in Figure 8, the proposed simplified model (as shown in Figure 5) of the single bridge-type mechanism was adopted to establish the operative bridge-type mechanisms I and II. However, the non-driven bridge-type mechanisms in the horizontal amplifiers are simplified as a mass-spring model, and the mass and stiffness of spring are equivalent to the output stiffness $k_{out}$ and mass $m_{b}$ of the single bridge-type mechanism, respectively. Similarly, the horizontal amplifiers in the *Y* direction are also a simplified mass-spring model, which is composed of a composite output stiffness $k_{out}/2$, an equivalent load stiffness $k_{loadx}$, and an equivalent lumped mass $m_{loadx}$. The composite output stiffness, $k_{out}/2$, is connected in series by the output stiffness of the two bridge-type mechanisms, but it has no hindrance effect on the motion in the *X* direction. However, the equivalent load stiffness $k_{loadx}$ has a complex hindrance effect on the *X* direction motion of the working platform, which is difficult to model and calculate directly, but which can be obtained by FEM simulation. In addition, all of the vertical amplifiers are simplified as a lumped mass element and integrated into the mass $m_{w}$ of the working platform. Due to the symmetric characteristics, the performance of the compliant platform system in the *Y* direction is the same as in the *X* direction. 

The output stiffness of the bridge-type mechanism can be generated as [16]:

(15) $$k_{out}=\frac{6EI}{l_{h}\left(l_{h}{}^{2}+3{\left(l_{h}+l_{l}\right)}^{2}\right)}\;$$

where *I* is the moment of inertia of flexure hinge which can be computed by $I=dt_{h}{}^{3}/12$.

**Figure 9:**
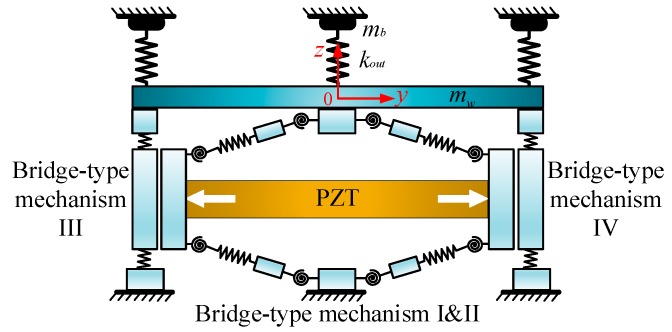
Mechanics model of the compliant platform in the Z direction.

In addition, the motion of the compliant platform that includes both the movement along the *Z* direction and rotation around the *X*/*Y* direction, can be represented through the mechanics model as shown in Figure 9. When the four bridge-type mechanisms at the bottom or top of the vertical amplifier are working together, movement along the *Z* direction can be achieved. The load stiffness is the output stiffness of the other four bridge-type mechanisms in the vertical amplifier in parallel, whose value is equal to $4k_{out}$ and it can be equivalent to a situation in which every working bridge-type mechanism takes an average output stiffness of the bridge-type mechanism. Thus, the mechanics model of the compliant platform movement along the *Z* direction can be equivalent when only one of the bridge-type mechanisms is working, and the equivalent load stiffness can be computed by: $k_{loadz}=k_{out}$. 

Similarly, when only one bridge-type mechanism at the bottom or top in the vertical amplifier is working, half of the rotary motion around the *X*/*Y* direction can be obtained. As two of the bridge-type mechanisms in the diagonal direction are working together, the entire rotary motion around the *X*/*Y* directions can be acquired during practical operation. The load stiffness $k_{loadrx}$ of rotation around the *X*/*Y* direction is t half of the coupled stiffness of the output stiffness of the other seven bridge-type mechanisms in the vertical amplifier, which can be obtained by FEM simulation. Thus, the mechanics characteristics of the compliant platform movement along the *Z* direction are the same as the rotation around the *X*/*Y* direction, except for the different load stiffness. 

**Figure 10:**
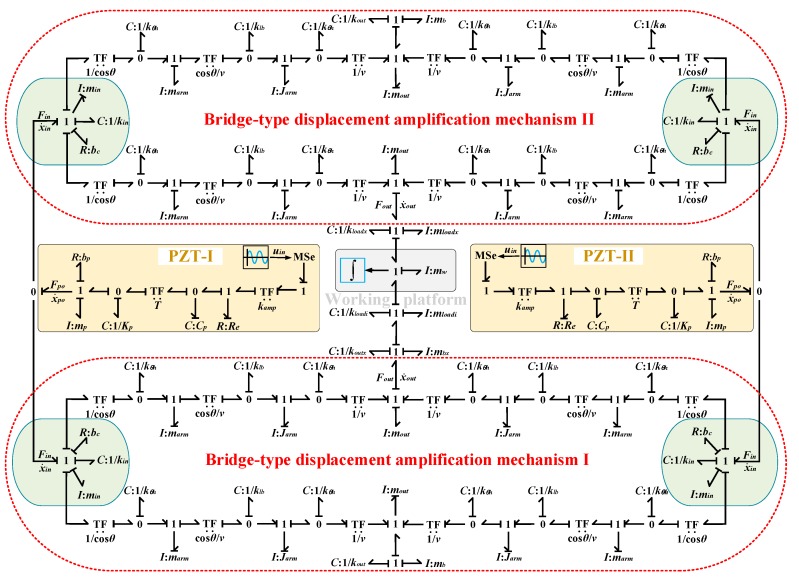
Bond graph model of the compliant platform system.

Based on the analysis above, by redefining some causality and simplifying the structure, the bond graph model of the compliant platform system, as established and shown in Figure 10, is general and valid for motion in all degrees of freedom. In Figure 10, the bridge-type mechanism I and bridge-type mechanism II work together to obtain movement along the *X*/*Y* direction. However, when only the bridge-type mechanism I with a different load stiffness is working, movement along the *Z* direction or rotation around the *X*/*Y* direction can be obtained. Meanwhile, the bond graph elements with the subscript *x* are not working in Figure 10. The subscript i=x,z,rx represents the corresponding parameters in the different motion directions. 

## 4. Results and Discussion

As shown in Figure 10, the bond graph model can be used to investigate the kinematic and dynamic characteristics of the proposed compliant platform system. In order to efficiently analyze the performances of the compliant platform system, the 20-Sim industrial simulation software has been adopted to model and simulate the bond graph model, which can easily derive and solve state space equations from the bond graph model as shown in Figure 10. The Vode Adams method was used to solve the state space equations, and the related parameters of the compliant platform are shown in Table 1 and Table 2. The output displacements of the working platform should be monitored in the simulation, and these are computed according to the following equation:

(16) $$X_{w}=\int v_{w}dt\;$$

**Figure 11:**
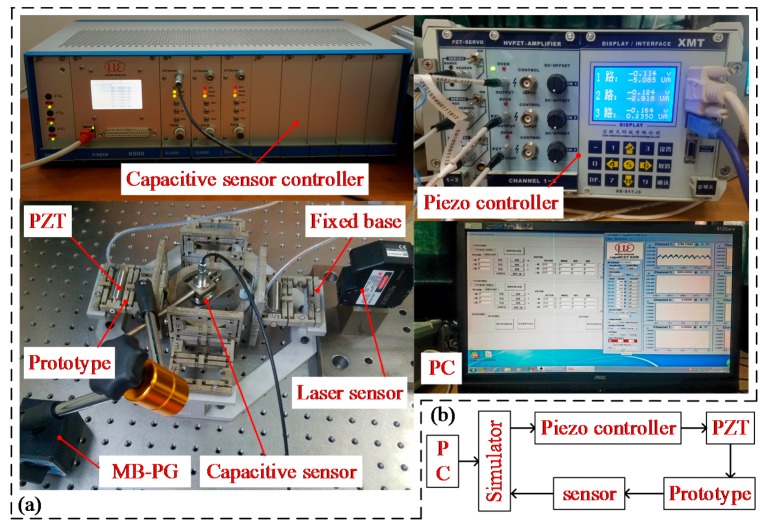
Experimental setup: (**a**) Experiment configuration; (**b**) experimental principle diagram of the compliant platform.

Furthermore, experimental tests were conducted to verify the effectiveness of the bond graph model of the proposed compliant platform. The prototype of the compliant platform was monolithically fabricated by a wire electrical discharge machining (WEDM) process, and the geometrical parameters of the compliant platform are listed in Table 1. The magnesium alloy AZ31b was selected as the material. The schematic diagram of the experimental setup is shown in Figure 11b. The testing experiments of the compliant platform were established as shown in Figure 11a, where all the fixed holes of the compliant platform were fixed on a fixed base that was mounted on an optical table to reduce the ground vibration. A piezo controller (model E01, from COREMORROW, Inc., Harbin, China) was utilized to drive the PZTs (model PSt-40VS15, from COREMORROW, Inc., Harbin, China). The PZTs with a nominal stroke of 38 μm at the driving voltage of 120 V, and other properties of the PZTs and drive circuit are listed in Table 2. The PZTs were inserted into the bridge-type mechanisms, and a preload was applied at the ends of PZT through two screws to ensure a proper and steady connection between both ends of the PZT and the bridge-type mechanism. The output displacements of the working platform were obtained by measuring the sensor target using a laser displacement sensor (model LK-H050, KEYENCE, Osaka, Japan) with a measurement range of 20 mm and a resolution of 100 nm. Meanwhile, the coupling displacements were measured by capacitance displacement sensors (model CS5, from MICRO-EPSILION, Inc., Bavaria, Germany) with a measurement range of 5 mm and a resolution of 100 nm.

**Figure 12:**
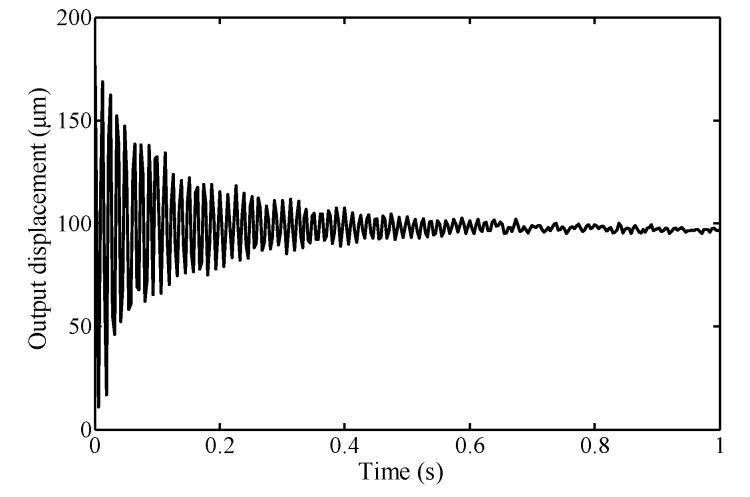
Response to step signal for system identification for the *X* direction.

To determine the dynamic parameters of the compliant platform experimentally, a step command signal with an amplitude of 50 V is generated and sent to the PZT in the *X* direction. The response of the compliant platform to step signal is measured and is shown in Figure 12.

The damping ratio ξ of this system can be estimated by using the percent overshoot of the system, which can be measured from the step response by finding the ratio of the maximum peak and steady state value:

(17) $$\xi =-\ln\left(M_{p}\right)/\sqrt{\pi^{2}+ln^{2}\left(M_{p}\right)}\;$$

where the percent overshoot is calculated as $M_{p}=78.39\%$, thus the damping ratio can be derived as ξ=0.08. The equivalent damping parameter $b_{c}$ can be derived by $b_{c}=2\xi \sqrt{k_{e}m_{e}}$ while the other equivalent parameters can be calculated by the following equations:

(18) $$\left\{ \begin{array}{l} k_{e}=k_{ouu}/4+2k_{loadx}\\ m_{e}=4m_{b}+2m_{loadx}+m_{w} \end{array}\right.\;$$

**Figure 13:**
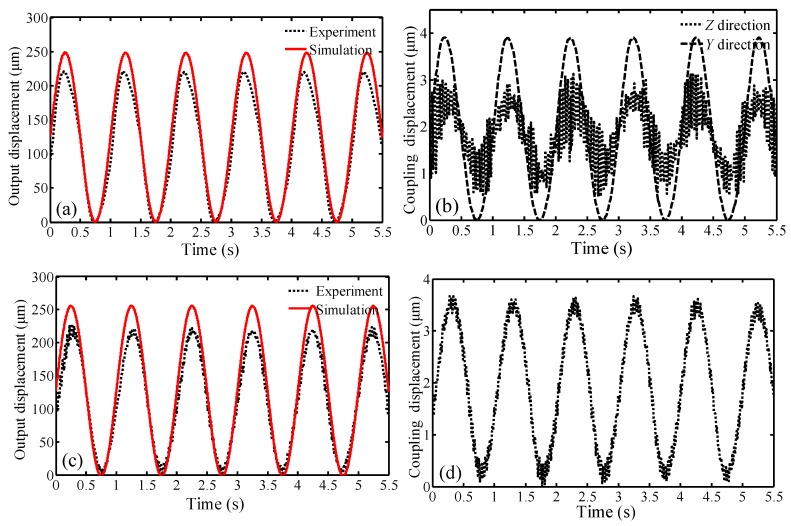

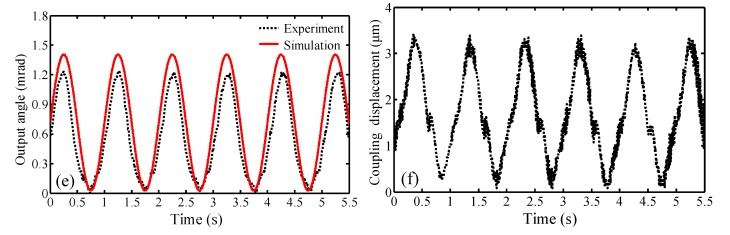
Simulated and experimental results of the compliant platform: (**a**) Output displacement in the *X* direction; (**b**) experimental coupling displacement in the *Y* and *Z* direction; (**c**) output displacement in the *Z* direction; (**d**) experimental coupling displacement in the *X* direction; (**e**) output angle around the *X* direction; (**f**) experimental coupling displacement in the *Y* direction.

In order to analyze the kinematic behaviors, the travel ranges of the compliant platform were obtained by the simulations and experiments. In addition, as the proposed compliant platform is designed symmetrically, the output coupling displacements were only measured in the experiments. For obtaining a maximum travel range, an amplitude of 120 V non-negative sinusoidal voltages signal with 1 Hz was input into the piezo controller to drive the PZT at each degree of freedom. The simulated and experimental results as well as the corresponding coupling results of each motion direction are illustrated in Figure 13 and listed in Table 3. 

From Figure 13a,c,e, it can be seen that the simulated results of the compliant platform movement along the *X* and *Y* directions, and rotation around the *X* direction, are consistent with the experimental results. According to Figure 13a–f, the maximum output displacements in the *X* direction of the simulation and experiment are 248.60 μm and 220.54 μm respectively, while the amplitude of experimental coupling displacement in the *Y* direction and *Z* direction are 3.91 μm and 3.05 μm respectively; therefore, the cross-axis coupling ratio in the *Y* direction and in the *Z* direction are 1.57% and 1.38% respectively. Similarly, the maximum output displacements in the *Z* direction of the simulation and experiment are 254.18 μm and 228.44 μm respectively, while the amplitude of experimental coupling displacement in the *X/Y* direction is 3.66 μm; therefore, the cross-axis coupling ratio in the *X* direction is 1.60%. In addition, the maximum output angle around the *X* direction of the simulation and the experiment are 1.40 mrad and 1.23 mrad respectively, while the amplitude of experimental coupling displacement in the *Y* direction is 3.43 μm; therefore, the cross-axis coupling ratio in the *Y* direction is 1.93%. According to the above analysis, the maximum coupling ratio is less than 2%, indicating the compliant platform has an excellent decoupling capability. The experimental coupling ratios may be attributed to many factors, such as the manufactured prototype lacking perfect symmetry, installation errors of the PZTs, inhomogeneity of the piezoelectric materials, and inherent noise from the capacitance sensors, etc. 

**Figure 14:**
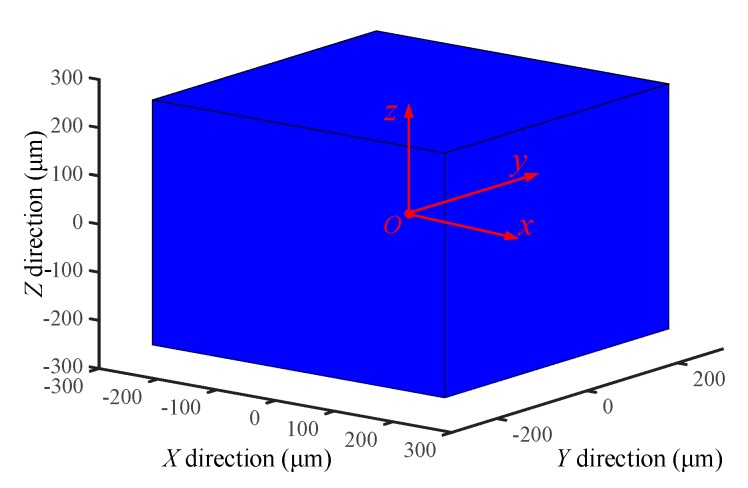
The reachable movement workspace.

As depicted in Figure 13a,c,e, the simulated values are slightly larger than the experimental values. In addition, the maximum errors between simulated results and experimental results for movement along the *X* and *Z-*axis, and rotation around the *X-*axis are 12.72%, 11.27% and 13.82%, respectively. These errors may be mainly attributed to: (1) in the bond graph modeling, the mechanical structures with minor deformation are assumed as the rigid body; (2) the mathematical description of the PZT is regarded as a linear model and the inherent nonlinearities such as hysteresis and creep are ignored in the bond graph modeling; (3) only the inverse piezoelectric effect of the piezoelectric material is considered, and the direct effect of the piezoelectric material is ignored. Because the deviation between the simulated results and experimental results is small, the correctness of the bond graph simulation is verified. Thus, the reliable maximum movement ranges of the proposed compliant platform can be predicted based on the simulated results. Due to the symmetrical structural design of the proposed compliant platform, the double movement ranges in the *X*, *Y* and *Z* directions are demonstrated in Figure 14.

For the flexure-based compliant platform, due to its high stiffness and small damping coefficient, vibration is a major factor affecting the performance of the compliant platform. The vibration will cause fluctuation of the output trajectory of the working platform. As discussed below, the vibration can be classified into two categories: (1) the inertial forced vibration is caused by the input excitations; (2) the high frequency component in the input signal excites the natural frequency of the compliant platform and causes mechanical resonances. 

Since the different input signals have a large effect on the dynamics responses, a step signal and a cycloidal step signal with amplitude of 50 V were respectively applied to the input end of the bond graph model to verify the dynamic performance of the compliant platform system in the *X* direction by simulation. The governing equation of cycloidal step signal is given as [40]:

(19) $$U\left(t\right)=\left\{ \begin{array}{l} U_{0}\left(\frac{t}{t_{r}}-\frac{1}{2\pi }\sin\left(2\pi \frac{t}{t_{r}}\right)\right)\;,\;t\leq t_{r}\\ U_{0}\;,\;t>t_{r} \end{array}\right.\;$$

where U(t) and $U_{0}$ are the output and amplitude of the cycloidal step signal respectively, and $t_{r}$ is the rise time of the cycloidal step signal. 

**Figure 15:**
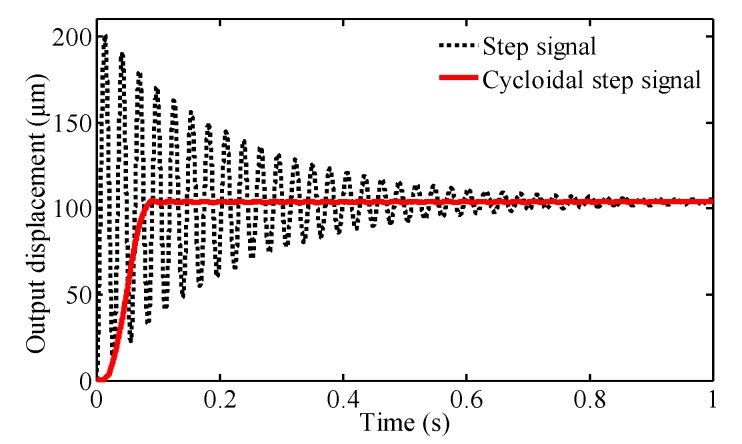
Responses of the compliant platform to different signals in the *X* direction.

As shown in Figure 15, because the mechanical structures are typically second-order dynamic systems with a small damping ratio as well as the infinite acceleration of response to step signal, there are large inertial vibrations resulting from large transient inertial force when the compliant platform is working. The overshoot is about 93% of the steady state value, and the settling time of the response to step signal is estimated as 0.92 s, indicting a low damping of the compliant platform with poor positioning accuracy and slow response speed. The compliant platform with the performance mentioned cannot be used for actual engineering applications. In Figure 15, it can be seen that the oscillation of the cycloidal step signal is almost eliminated when the rising time is set as 0.1 s, and the overshoot is less than 0.5% of the vibration equilibrium displacement. Thus, the cycloidal step signal can improve the dynamic performance of the compliant platform. By comparing Figure 12 and Figure 15, the equilibrium displacements of experimental response to step signal are slightly less than the simulated results. The main reason for this is that the extra components, such as the PZT, were inserted in the bridge-type mechanism, and the sensor targets were attached at the working platform, and the extra load mass will influence the dynamics performance of the compliant platform.

**Figure 16:**
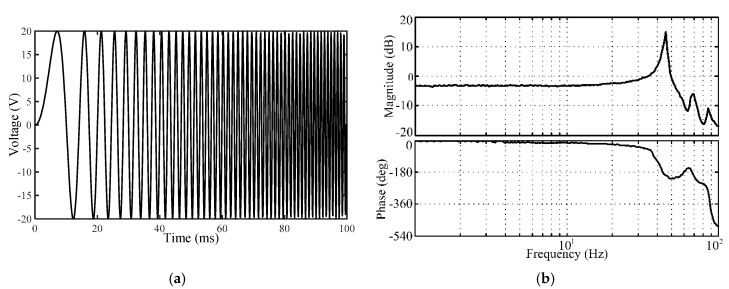
Frequency response of the compliant platform: (**a**) Sweep signal waveform; (**b**) frequency response along the *X/Y*-axis.

In order to avoid the mechanical resonances, frequency response analysis was carried out with the aid of the frequency domain toolbox of software 20-sim to evaluate the natural frequency of the compliant platform. As shown in Figure 16a, the resonant frequency of the compliant platform movement in the *X*/*Y* is simulated using a sine sweep signal with amplitude of 50 V, ranging from 1 to 100 Hz. Figure 16b shows the results of the *X/Y* direction only, showing its natural frequency of 45.46 Hz. Similarly, the frequency responses in the *Z* direction and around the *X*/*Y* direction are identified by adopting two swept excitation signals, respectively. The natural frequency of 57.25 Hz in the *Z* direction and 99.83 Hz around the *X*/*Y* are obtained. Furthermore, frequency response testing experiments are also carried out to verify the dynamic performance of the compliant platform. The natural frequencies of 41 Hz and 52 Hz along the *X*/*Y* and *Z* directions, and 87 Hz around the *X*/*Y* direction are identified, respectively. It can be seen that the relative deviations of the simulated frequencies compared with the experimental frequencies are 10.87%, 10.10%, and 14.75% along the *X/Y*, *Z* directions and around the *X/Y* direction, respectively.

**Figure 17:**
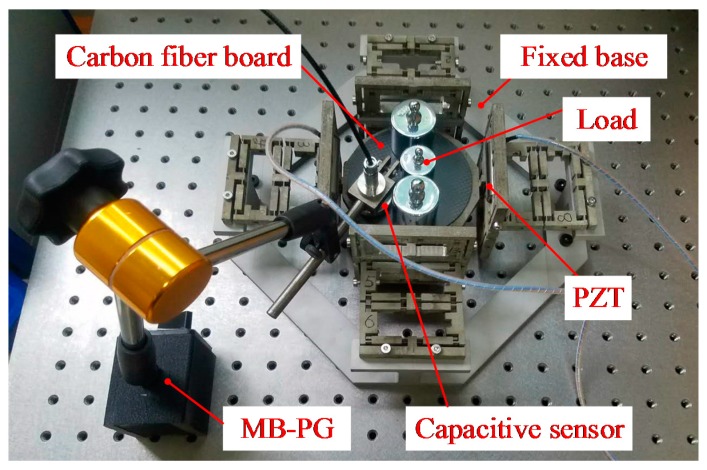
Experimental setup for load capacity tests.

**Figure 18:**
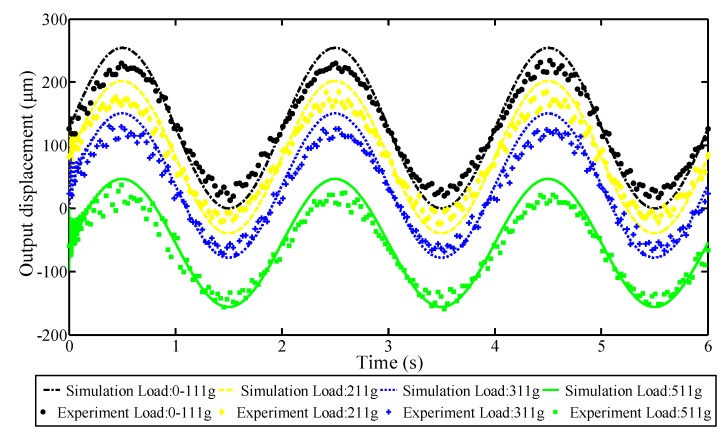
Responses of the compliant platform with various loads to harmonic signals.

The load capacity is an important performance indicator for the compliant mechanism. During operation, the direction of load always points toward the negative direction of the *Z*-axis, which will hinder the translational motion of the working platform in the positive direction of the *Z*-axis. In order to evaluate the load capacity of the compliant platform in a vertical direction, the travel ranges of the compliant platform with different constant load conditions were obtained through simulation and experiment. In the simulation, an amplitude of 120 V sinusoidal signal with 0.5 Hz was applied, and a constant load element was connected with the inertial element of the working platform through a 1-junction, which is a common flow function in bond graph theory. As shown in Figure 17, the experimental setup for load capacity tests in the *Z* direction was established, where a carbon fiber board with the weight of 11 g was glued on the working platform as the support platform for various loads. The simulated results and testing results that were measured by a capacitance sensor are presented in Figure 18.

As demonstrated in Figure 18, the simulated results are in good agreement with the experimental results. These results prove that the bond graph is an effective way to study the load capacity for the compliant platform. Moreover, it can be seen that when the load is less than 100 g, the simulated results are the same as the non-loaded results. But when the load value reaches 200 g, the simulated results are obviously less than the non-loaded results, indicating that the maximum load capacity in the *Z* direction is less than 200 g. In addition, the strokes of the working platform in the positive direction of the *Z*-axis are reduced from 254.19 μm to 46.67 μm when the load is increased from 0 g to the 500 g, which results from the small output stiffness of the bottom bridge-type mechanisms in the vertical amplifier. In addition, as depicted in Figure 18, the high frequency fluctuation at the beginning of the simulated curve is mainly caused by the initial condition, which sets the load on working platform as a constant value. 

## 5. Conclusions

This paper presents a bond graph model of a piezoelectric-actuated micro-/nano compliant platform system, and explores a general approach to investigate the kinematic and dynamic characteristics of the compliant platform system. The proposed bond graph model is constructed by combining the piezoelectric actuator subsystem and compliant platform subsystem. Based on the energy flow relationship of the inverse hysteresis effect of the piezoelectric actuators, the piezoelectric actuators are divided into the electric domain and mechanical domain, and then a bond graph model of the piezoelectric actuator is generated by coupling the two kinds of domains in the modeling. Specifically, the bond graph model for bridge-type mechanism is established based on pseudo-rigid-body (PRB) model theory and elastic beam theory, which consider the compliances of flexure hinges, elastic linkages and input ends. The complete bond graph model of the proposed compliant platform system is constructed by coupling the piezoelectric actuator subsystem and compliant platform subsystem.

In order to investigate the kinematic and dynamic performance of the proposed compliant platform system, both simulations and experiments for this system are conducted. The simulated results and experimental results are comparatively analyzed. The maximum average error of travel ranges between simulated values and experimental values is 13.82%, by which the correctness of bond graph model is verified. Meanwhile, the experimental results indicate that the cross-axis coupling error is evaluated to be below 2%, indicating the proposed compliant platform has an excellent decoupling capacity. Furthermore, the simulated results reveal that the cycloidal step signal can improve the dynamic performance of the compliant platform. The frequency responses and load capacity have been investigated through both computer simulations and experimental tests. In the future, the proposed compliant platform system has wide potential applications in precision engineering applications, such as nanopositioning systems and optical alignment systems. Based on the identified kinematic and dynamic characteristics, future research will focus on controller design to reduce the cross-axis coupling errors and minimize the inherent nonlinear errors of the piezoelectric actuators, and vibration control to compensate for the vibration errors of the compliant platform.

## Figures and Tables

**Table 1:** Material and mechanical parameters of the compliant platform.

Items	Parameters	Values	Parameters	Values
Material:AZ31b	Young’s modulus *E* (GPa)	45	Density *ρ* (kg/m^3^)	1780
	Poisson’s ratio μ	0.34	Yield strength $\sigma_{S}$ (MPa)	288
Bridge-type mechanism	$l_{h}$ (mm)	4	$l_{l}$ (mm)	20
	t (mm)	0.8	h (mm)	5
	v (mm)	2.2	d (mm)	8
	$l_{in}$ (mm)	21.8	*b* (mm)	8
Compliant platform	$m_{in}$ (kg)	3.019 × 10^−3^	$m_{loadx}$ (kg)	0.021
	$m_{out}$ (kg)	4.272 × 10^−4^	$k_{loadx}$ (N/mm)	3.251
	$m_{arm}$ (kg)	1.520 × 10^−3^	$m_{loadz}$ (kg)	0.048
	$m_{w}$ (kg)	0.140	$k_{loadz}$ (N/mm)	13.21
	$m_{b}$ (kg)	0.013	$m_{loadrx}$ (kg)	0.171

**Table 2:** Specifications of the piezoelectric actuator and the drive circuit.

Items	Properties	Values	Properties	Values
PZT (PSt-40VS15)	Dimensions (mm)	Φ15 × 50	Axis stiffness $K_{p}$ (N·μm^−1^)	60 ± 20%
	*n*	360	Thickness Δ*l* (μm)	100
	Maximum driving force (N)	2300	Transformation factor *T* (N/V)	19.167
	Capacitance c (μF)	2.5	Viscous damping $b_{p}$ (N·s·m^−1^)	150
	Resonant frequency (kHz)	20	Mass $m_{p}$ (kg)	0.04
Driver circuit	Resistance $R_{e}$ (Ω)	280	Amplification ratio $k_{amp}$	15

**Table 3:** Analysis results of the compliant platform.

Items	Maximal Movement Stroke (μm)			Maximal Rotation Stroke (mrad)
	*X* direction		*Z* direction	*X* direction
Experiment	220.54		228.44	1.23
Simulation	248.60		254.18	1.40
Average Error (%)	12.72		11.27	13.82
	*Y* direction	*Z* direction	*X*/*Y* direction	*Y* direction
Coupling Displacement (μm)	3.91	3.05	3.66	3.43
Coupling Ratio (%)	1.57	1.38	1.60	1.93
